# Temporal changes in mammographic breast density and breast cancer risk among women with benign breast disease

**DOI:** 10.1186/s13058-024-01764-2

**Published:** 2024-03-26

**Authors:** Maeve Mullooly, Shaoqi Fan, Ruth M. Pfeiffer, Erin Aiello Bowles, Máire A. Duggan, Roni T. Falk, Kathryn Richert-Boe, Andrew G. Glass, Teresa M. Kimes, Jonine D. Figueroa, Thomas E. Rohan, Mustapha Abubakar, Gretchen L. Gierach

**Affiliations:** 1 School of Population Health, RCSI University of Medicine and Health Sciences, Dublin, Ireland; 2 Division of Cancer Epidemiology and Genetics, National Cancer Institute, Bethesda, MD USA; 3 Kaiser Permanente Washington Health Research Institute, Kaiser Permanente Washington, Seattle, WA USA; 4 Department of Pathology and Laboratory Medicine, University of Calgary, Calgary, AB T2N2Y9 Canada; 5 Kaiser Permanente Center for Health Research, Portland, OR USA; 6 Usher Institute, University of Edinburgh, Edinburgh, UK; 7 Department of Epidemiology and Population Health, Albert Einstein College of Medicine, Bronx, NY USA

**Keywords:** Mammographic breast density, Breast cancer risk, Benign breast disease

## Abstract

**Introduction:**

Benign breast disease (BBD) and high mammographic breast density (MBD) are prevalent and independent risk factors for invasive breast cancer. It has been suggested that temporal changes in MBD may impact future invasive breast cancer risk, but this has not been studied among women with BBD.

**Methods:**

We undertook a nested case–control study within a cohort of 15,395 women with BBD in Kaiser Permanente Northwest (KPNW; 1970–2012, followed through mid-2015). Cases (*n* = 261) developed invasive breast cancer > 1 year after BBD diagnosis, whereas controls (*n* = 249) did not have breast cancer by the case diagnosis date. Cases and controls were individually matched on BBD diagnosis age and plan membership duration. Standardized %MBD change (per 2 years), categorized as stable/any increase (≥ 0%), minimal decrease of less than 5% or a decrease greater than or equal to 5%, was determined from baseline and follow-up mammograms. Associations between MBD change and breast cancer risk were examined using adjusted unconditional logistic regression.

**Results:**

Overall, 64.5% (*n* = 329) of BBD patients had non-proliferative and 35.5% (*n* = 181) had proliferative disease with/without atypia. Women with an MBD decrease (≤ − 5%) were less likely to develop breast cancer (Odds Ratio (OR) 0.64; 95% Confidence Interval (CI) 0.38, 1.07) compared with women with minimal decreases. Associations were stronger among women ≥ 50 years at BBD diagnosis (OR 0.48; 95% CI 0.25, 0.92) and with proliferative BBD (OR 0.32; 95% CI 0.11, 0.99).

**Discussion:**

Assessment of temporal MBD changes may inform risk monitoring among women with BBD, and strategies to actively reduce MBD may help decrease future breast cancer risk.

**Supplementary Information:**

The online version contains supplementary material available at 10.1186/s13058-024-01764-2.

## Introduction

In the United States, more than 70% of 1.6 million annual breast biopsies are benign, with benign breast disease (BBD) collectively representing a heterogeneous group of pathologically defined breast lesions [1]. BBD is associated with increased breast cancer risk, and the strength of the risk association varies according to the severity of the histological changes observed on the biopsy lesion [2, 3]. Meta-analysis shows that women with atypical hyperplasia, the most severe form of BBD, have an almost fourfold increased risk of breast cancer development [2].

Mammographic breast density (MBD) is the radiological description of fibrous and glandular (fibroglandular) tissue on a mammogram [4]. Elevated MBD is associated with a four-to-sixfold higher breast cancer risk compared with women with the lowest MBD levels [4]. Prior work has investigated associations between the tissue composition of BBD with MBD and has demonstrated that MBD is associated with histologic tissue composition metrics, including epithelial nuclear area and connective tissue stroma [5–7]. In a recent analysis of this study population, we validated associations between MBD and quantitative tissue compositions metrics and also found a strong inverse correlation between MBD and quantitative variations in the proportion of fibroglandular tissue that is epithelium relative to connective tissue stroma (i.e., epithelium-to-stroma proportion (ESP)). Increasing ESP was associated with elevated risk of future invasive breast cancer and the combination of high ESP and high MBD identified a subset of women at greatly elevated risk of breast cancer [8]. The findings were also suggestive of low MBD attenuating the risk associated with high ESP and vice versa, particularly among women with proliferative disease. MBD is a dynamic trait that changes over a woman’s lifetime and both endogenous and exogenous exposures have been associated with changes in MBD and mammary tissue composition. Physiologically, MBD is higher among younger, premenopausal women and decreases with advancing age [9]. Other factors such as parity, menopause and elevated BMI [10, 11] are reportedly associated with decreases in MBD, while menopausal hormone therapy (MHT) use has been linked to increases in MBD [12–14]. However, it is not known whether such temporal changes may impact risk of future breast cancer among BBD patients.

Few observational studies have examined associations between temporal changes in MBD and subsequent breast cancer risk but these have largely been conducted among women of average risk and have reported inconsistent findings [15–20]. To our knowledge, no study has examined temporal changes and future breast cancer risk among women with BBD, a group at higher breast cancer risk. For women with BBD, strategies are needed for monitoring risk and for prevention of future cancer. Should temporal declines in MBD be associated with reduced breast cancer risk, actions that promote these temporal declines, and/or include evaluation of temporal changes in MBD, may be beneficial as part of the management of women diagnosed with BBD. In a cohort of women diagnosed with BBD within an integrated healthcare system, this study aimed to examine whether temporal changes in MBD experienced among women with BBD were associated with subsequent breast cancer risk.

## Methods

### Overview of study population

This study used a case–control design nested within a cohort of women diagnosed with BBD within the Kaiser Permanente Northwest Region (KPNW) healthcare system [21]. The cohort included women (*n* = 15,395; aged 21–85 years) who were diagnosed with BBD between 1970 and 2012 and followed up through mid-2015 [21]. As previously described [8], cases (*n* = 514) included women who had a BBD diagnosis and subsequently developed invasive breast cancer at least one year after the index BBD biopsy. Controls (*n* = 514) were women selected using a risk-set sampling approach from the KPNW cohort and were individually matched to each case on age at BBD diagnosis (+ /−1 year) and plan membership duration and did not have a breast cancer before the matched case’s diagnosis date. The current study population was restricted to those diagnosed with BBD in 1980 or later (*n* = 261 cases and *n* = 249 controls) to ensure that all mammograms were based on film screening mammography (i.e., excluding xeromammography).

### Risk factor information

Information on breast cancer risk factors for the study population was collected from KPNW medical records. Demographic and reproductive information available included BBD histology (normal/non-proliferative, proliferative with/without atypia), age at BBD diagnosis (< 50, 50–59, ≥ 60 years), parity/age at first live birth (nulliparous/age at first live birth ≥ 30 years, parous/age at first live birth < 30 years), family history of breast cancer in a first degree relative (absent, present), and [8] combined menopausal status and menopausal hormone therapy (MHT) use (premenopausal, postmenopausal and using MHT, postmenopausal no MHT use, postmenopausal unknown MHT use). Body mass index (BMI) at time of biopsy (< 25, 25–29, ≥ 30 kg/m^2^) was also included; however, BMI at time of follow-up mammogram was not available for inclusion as a covariate in the analysis.

### Breast tissue histology

Archival representative formalin-fixed paraffin-embedded tissue blocks from the index BBD biopsy were retrieved and sectioned, and a hematoxylin and eosin (H&E) stained section was prepared for histological review. The BBD H&E slides were reviewed centrally for diagnosis. In addition, all histological diagnoses were reviewed by study pathologists (MAD and MA) and appropriately classified according to the published Dupont and Page criteria [22]. The corresponding H&E-stained slides were centrally reviewed and classified as normal/non-proliferative, proliferative without atypia, and atypical hyperplasia. Terminal duct lobular unit (TDLU) involution was assessed and impression of involution was categorized according to the Mayo BBD cohort visual assessment criteria [23] as none/mild involution (0–24% of TDLUs involuted), partial involution (25–74%), complete involution (≥ 75%) or no TDLUs observed.

Additionally, as previously described [8], quantitative data on the amount of epithelium, stroma, and adipose tissue on the breast biopsy were obtained by using supervised machine learning algorithms (Halo 1.2 Tissue Classifier algorithm, Indica Labs, Albuquerque, New Mexico). Briefly, H&E-stained slides were scanned using the Aperio digital slide scanner at 20X (Leica Biosystems Inc. Buffalo Grove, IL) and a 22 datapoint script was developed to identify, segment and quantify epithelium, stroma, and adipose tissue within each H&E slide. For this current analysis, the percent of each of the three tissue components within the total tissue area of the slide was determined and categorized as low or high according to the median value of the control group. In addition, the epithelium-to-stroma proportion was calculated as a percentage, (epithelial area / total epithelial and stromal area) × 100, and categorized as low or high according to the median value of the study control group.

### Retrieval of mammograms and mammographic breast density assessment

Baseline craniocaudal mammograms of the breast ipsilateral (preferable; 88.8%) or contralateral (11.2%) to the BBD biopsy were selected closest to and preferably before the BBD biopsy date. Where possible, mammograms within 6 months before the BBD biopsy date were selected. Where no pre-biopsy mammogram was available, a mammogram within 1 month after the BBD biopsy date was retrieved. Fifty-three percent of the population (58.2% of cases and 49.8% of controls) had mammograms retrieved within 1 month before or after the BBD diagnosis date. For the follow-up time-point, a second mammogram of the same baseline laterality, closest to and up to or before the date of breast cancer diagnosis (cases) or selection (controls) was retrieved. If not available, a mammogram as close to the diagnosis/selection date, where possible on or within approximately 1 month after, was chosen. Forty-eight percent of the population had follow-up mammograms retrieved within 1 month before or after the breast cancer diagnosis/selection date. Among the cases, only four (0.8%) cases had follow-up mammograms obtained > 1 month post-breast cancer diagnosis date.

For the assessment of MBD, craniocaudal film mammographic views were digitized using an Array Corporation 2095 Laser Film Digitizer (Roden, the Netherlands; optical density = 4.0). The interactive computer-assisted thresholding program, Cumulus^®^, was used to estimate quantitative breast density measures [24]. For all mammograms (baseline and follow-up), absolute dense area (cm^2^), total breast area (cm^2^) and percent density ((absolute dense area / total breast area) × 100) were estimated by a single expert mammogram reader (EAB). Baseline and follow-up mammographic images from cases and matched controls were read in the same batch in random order. A repeat set of approximately 10% (*n* = 113) images demonstrated excellent reproducibility as previously reported [8]. MBD change was defined as the standardized change in percent density per 2 years, according to the following formula: MBD change per 2 years = (percent density at follow-up mammogram − percent density at baseline mammogram)/(calendar years between baseline mammogram and follow-up mammogram/2).

### Statistical analysis

Standardized change in percent MBD per 2 years was categorized according to the following cut-points: stable/any increase greater than or equal to 0% MBD change per 2 years (≥ 0%); minimal decrease of less than 5% MBD change per 2 years (5% < to < 0) and a decrease greater than or equal to 5%. We combined those whose density remained stable or increased within the same category, based on the hypothesis that these groups represent departures from the expected physiological process of age-related temporal declines in MBD. Associations between categories of MBD change and breast cancer risk factors were examined using chi-squared tests, both overall and by case–control status. As previously described [8, 25], multiple imputation implemented in IVEware (http://www.isr.umich.edu/src/smp/ive) was performed for the full cohort, to impute missing risk factor data, for covariates with < 40% missing data. Imputed variables included age at menarche, parity and age at first birth, body mass index, family history, menopausal and MHT use, bilateral oophorectomy, age at BBD, parity, number of pregnancies, and history of hysterectomy. Only age at menarche had more than 20% missing data with the remaining variables having < 20% missingness. An overview of missing data and the multiple imputation approach is provided in Figueroa et al [25] and Abubakar et al. [8].

Given that mammograms could not be retrieved for all of the original selected case and control matched sets, we broke the matched design and used unconditional logistic regression models to investigate the associations between MBD change and invasive breast cancer. In the primary analysis, women with stable/increased or decreased density were compared to those with minimal decreases using multivariable unconditional logistic regression and adjusted for matching factors (continuous age at BBD, continuous follow-up time between BBD and breast cancer diagnosis). Models were also adjusted for categorical BBD calendar year diagnosis (1980–1989, 1990–1999, 2000–2011), categorical BMI and quartiles of baseline MBD (based upon the control distribution). Given that prior work has demonstrated substantial declines in MBD over the menopausal transition, analyses were stratified by age at BBD diagnosis (< 50 and ≥ 50 years) as a surrogate for menopausal status. Further subgroup analysis stratified by BBD histological classification (normal/non-proliferative, proliferative with/without atypia) was also conducted. In sensitivity analysis, the primary analysis was repeated for the matched case–control sets (*n* = 189) for which mammograms could be retrieved, using conditional logistic regression. We assessed statistical interactions of MBD change and follow-up time (continuous), BMI (categorical), age at BBD diagnosis (< 50 vs. ≥ 50 years), BBD histology and histologic composition (categorical) on the BBD H&E, using likelihood ratio tests. All statistical analyses were carried out using SAS 9.4. *P* values of < 0.05 were considered statistically significant and all tests were two-tailed.

## Results

### Characteristics of study population

The analytical population comprised 510 BBD patients of whom cases (*n* = 261) developed invasive breast cancer > 1 year after BBD diagnosis, whereas controls (*n* = 249) did not have breast cancer by the case diagnosis date. BBD histology and % MBD at baseline varied by breast cancer case–control status. Among the cases, 57.9% (*n* = 151) had a BBD diagnosis of non-proliferative histology, with the remaining 42.1% (*n* = 110) having a diagnosis of proliferative disease with or without atypia. Among the controls, 71.5% (*n* = 178) had a diagnosis of non-proliferative BBD, whereas 28.5% (*n* = 71) had a BBD diagnosis of proliferative histology. The mean (standard deviation [SD]) baseline percent MBD was 32.6% (18.8%), with greater baseline density observed among cases (mean = 34.1%, SD: 18.2%) than controls (mean = 31.0%, SD: 19.3%).

The mean (SD) time between baseline mammogram and BBD diagnosis was − 1.2 (2.8) months for cases and − 1.3 (4.3) months for controls. The mean (SD) time between baseline mammogram and follow-up mammogram was 8.7 (5.5) years for cases and 8.3 (5.4) years for controls. The mean (SD) time between follow-up mammogram and breast cancer or corresponding selection date was − 1.8 (9.5) months for the cases and − 2.1 (6.7) months for the controls, respectively.

The distributions of risk factors across categories of MBD change by case–control status are shown in Table 1. Among the total study participants, 19% (97/510) experienced a decrease greater than or equal to 5% (≥ 5%) in MBD per 2 years, 55.5% (283/510) had a minimal decrease of less than 5% MBD change per 2 years (0 to  < 5% per 2 years) and 25.5% (130/510) experienced stable/any increase in MBD during follow-up. Among controls, women were younger at BBD diagnosis (*p* = 0.006), had a lower BMI (*p* = 0.008) and had higher baseline MBD (*p* < 0.0001) and were more likely to experience MBD decline. No associations with MBD change were observed for BBD histology, parity/age at first live birth, and family history (Table 1).

**Table 1:** Association between standardized percent mammographic breast density change and breast cancer risk factors by case–control status

Characteristic	Cases (*n* = 261)						Controls (*n* = 249)					
	%	Baseline MBD Mean (Range)	Stable/any increase (*n* = 65, 24.9%) *N* (%)	Minimal decrease (*n* = 151, 57.9%) *N* (%)	Decrease (n = 45, 17.2%) *N* (%)	*p*-value*	%	Baseline MBD Mean (Range)	Stable/any increase (*n* = 65, 26.1%) *N* (%)	Minimal decrease (*n* = 132, 53.0%) *N* (%)	Decrease (*n* = 52, 20.9%) N (%)	*p*-value*
*BBD histotype*												
Normal/Non-proliferative	57.9	33.6 (2.3, 83.7)	40 (61.5)	85 (56.3)	26 (57.8)	0.77	71.5	31.8 (2.4, 82.3)	43 (66.2)	100 (75.8)	35 (75.8)	0.28
Proliferative diagnosis (with/out atypia)	42.1	34.7 (3.7, 81.6)	25 (38.5)	66 (43.7)	19 (42.2)		28.5	29.1 (0.2, 75.0)	22 (33.9)	32 (24.2)	17 (24.2)	
*Age at BBD (years)*												
< 50	36.0	42.1 (8.3, 83.7)	13 (20.0)	62 (41.1)	19 (42.2)	** < 0.0001**	31.7	37.7 (3.4, 77.6)	13 (20.0)	53 (40.2)	13 (40.2)	**0.0062**
50–59	34.5	34.1 (2.7, 79.7)	18 (27.7)	58 (38.4)	14 (31.1)		35.7	30.8 (5.2, 82.3)	21 (32.3)	45 (34.1)	23 (34.1)	
≥ 60	29.5	24.2 (2.3, 62.3)	34 (52.3)	31 (20.5)	12 (26.7)		32.5	24.8 (0.2, 79.4)	31 (47.7)	34 (25.8)	16 (25.8)	
Median (range)		54.2 (32.6, 86.6)	61.3 (35.9, 86.6)	52.7 (32.6, 78.6)	51.8 (36.2, 78.5)			55.0 (32.4, 86.2)	59.8 (32.4, 86.2)	52.3 (36.0, 78.7)	55.0 (38.8, 73.6)	
*Parity/age at first live birth (ALFB)*												
Nulliparous/AFLB ≥ 30 years	23.7	40.2 (2.7, 81.6)	12 (19.1)	38 (25.0)	12 (25.8)	0.54	22.7	37.9 (0.2, 82.3)	12 (18.2)	33 (25.2)	11 (21.9)	0.58
Parous/AFLB < 30 years	76.3	32.2 (2.3, 83.7)	53 (80.9)	113 (75.0)	33 (74.2)		77.3	29.0 (1.1, 79.4)	53 (81.8)	99 (74.8)	41 (78.1)	
*BMI at biopsy (kg/m*^*2*^*)*												
< 25	35.5	43.5 (2.3, 83.7)	20 (30.8)	58 (38.4)	14 (31.1)	0.69	37.3	41.1 (4.9, 82.3)	19 (29.2)	55 (41.7)	19 (36.5)	**0.0083**
25–29	36.6	30.1 (3.7, 74.4)	28 (43.1)	51 (33.8)	18 (40.0)		33.5	27.8 (0.2, 74.6)	16 (24.6)	46 (24.9)	22 (42.3)	
≥ 30	28.0	27.3 (2.7, 81.6)	17 (26.2)	42 (27.8)	13 (28.9)		29.2	21.8 (1.1, 64.9)	30 (46.2)	31 (23.5)	11 (21.2)	
*Family history*												
Absent	77.9	34.5 (2.7, 83.7)	47 (72.0)	120 (79.2)	37 (81.8)	0.41	82.6	31.3 (0.2, 82.3)	58 (88.6)	107 (81.4)	41 (78.1)	0.31
Present	22.1	32.7 (2.3, 81.6)	18 (28.0)	31 (20.8)	8 (18.2)		17.4	29.4 (3.7, 79.4)	7 (11.4)	25 (18.6)	11 (21.9)	
*Menopause and MHT use*												
Premenopausal	38.5	41.2 (6.6, 83.7)	14 (21.5)	69 (45.7)	18 (40.0)	–	32.8	38.3 (3.4, 77.6)	14 (21.5)	53 (40.2)	15 (28.9)	0.16
Postmenopausal MHT use	43.8	29.7 (2.3, 72.5)	38 (58.5)	57 (37.8)	18 (40.0)		53.2	28.6 (1.1, 82.3)	38 (58.5)	64 (48.5)	30 (57.7)	
Postmenopausal No MHT	0.8	15.0 (7.7, 22.2)	0	1 (0.7)	1 (2.2)		4.1	22.1 (4.9, 37.7)	4 (6.2)	5 (3.8)	1 (1.9)	
Postmenopausal Unknown MHT	16.9	29.9 (2.7, 79.7)	13 (20.0)	24 (15.9)	8 (17.8)		10.0	23.6 (0.2, 57.1)	9 (13.9)	10 (7.6)	6 (11.5)	
*Impression of involution*												
None/mildly involuted (0–24%)	57.5	35.3 (4.0, 79.7)	22 (44.)	87 (64.9)	18 (47.4)	**0.016**	47.6	33.3 (3.4, 77.6)	21 (45.7)	57 (50.0)	20 (43.5)	0.18
Partially involuted (25–74%)	19.9	36.0 (8.3, 74.4)	9 (18.4)	23 (17.2)	12 (31.6)		18.5	37.8 (8.5, 82.3)	7 (15.2)	17 (14.9)	14 (30.4)	
Completely involuted (≥ 75%)	22.6	33.6 (2.3, 83.7)	18 (36.7)	24 (17.9)	8 (21.1)		34.0	29.5 (2.4, 69.2)	18 (39.1)	40 (35.1)	12 (26.1)	
No TDLU observed		28.4 (2.7, 81.6)	16	17	7			22.1 (0.2, 70.4)	19	18	6	
*Epithelial-to-Stroma proportion*												
Low	37.1	36.6 (6.3, 83.7)	22 (36.1)	57 (39.3)	12 (30.8)	0.61	49.8	31.7 (0.2, 75.0)	30 (48.4)	68 (54.8)	19 (54.8)	0.16
High	62.9	32.4 (2.3, 81.6)	39 (63.9)	88 (60.7)	27 (69.2)		50.2	30.2 (1.1, 82.3)	32 (51.6)	56 (45.2)	30 (45.2)	
*Epithelium*												
Low	43.3	31.6 (2.3, 83.7)	28 (45.9)	63 (43.5)	15 (38.5)	0.76	51.1	29.1 (0.2, 70.4)	34 (54.8)	60 (48.4)	26 (48.4)	0.67
High	56.7	35.7 (3.7, 81.6)	33 (54.1)	82 (56.6)	24 (61.5)		48.9	32.9 (1.1, 82.3)	28 (45.2)	64 (51.6)	23 (51.6)	
*Stroma*												
Low	48.2	28.2 (2.3, 73.6)	30 (49.2)	70 (48.3)	18 (46.2)	0.96	49.8	26.6 (0.2, 82.3)	37 (59.7)	51 (41.1)	29 (41.1)	**0.020**
High	51.8	39.3 (5.0, 83.7)	31 (50.8)	75 (51.7)	21 (53.9)		50.2	35.3 (1.6, 77.0)	25 (40.3)	73 (58.9)	20 (58.9)	
*Adipose tissue*												
Low	55.9	38.9 (5.0, 83.7)	32 (52.5)	81 (55.9)	24 (61.5)	0.67	50.2	35.2 (1.1, 79.4)	27 (43.6)	69 (55.7)	22 (55.7)	0.21
High	44.1	27.7 (2.3, 63.2)	29 (47.5)	64 (44.1)	15 (38.5)		49.8	26.7 (0.2, 82.3)	35 (56.5)	55 (44.4)	27 (44.4)	
*Baseline MBD (%)*												
*Q*1: ≤ 15.1	16.5	–	24 (36.9)	17 (11.3)	2 (4.4)	** < 0.0001**	25.3	–	35 (53.9)	27 (20.5)	1 (1.9)	** < 0.0001**
*Q*2: 15.1 < to ≤ 28.9	26.8	–	22 (33.9)	41 (27.2)	7 (15.6)		24.9	–	18 (27.7)	34 (25.8)	10 (19.2)	
*Q*3: 28.9 < to ≤ 43.6	27.2	–	10 (15.4)	47 (31.1)	14 (31.1)		24.9	–	7 (10.8)	36 (27.3)	19 (36.5)	
*Q*4: >43.6	29.5	–	9 (13.9)	46 (30.5)	22 (48.9)		24.9	–	5 (7.7)	35 (26.5)	22 (42.3)	

Mean (range) baseline percent density in all women = 32.6 (0.2, 83.7); among cases = 34.1 (2.3, 83.7); among controls = 31.0 (0.2, 82.3)

Standardized change in percent mammographic density (i.e., percent density change per two years) was calculated using percent density change from baseline to follow-up mammogram and divided by time intervals between two mammogram exams  [(percent density at follow-up mammogram − percent density at baseline mammogram)/(calendar years between baseline mammogram and follow-up mammogram/2)]

Missing values in breast cancer risk factors were imputed using multiple imputations. Result estimates were calculated in five imputed datasets, separately, and averaged using statistical procedures

*AFLB age at first live birth, BBD* benign breast disease, *BMI* body mass index, *CI* confidence interval, *MBD* mammographic breast density, *OR* odds ratio, *Q* quartile

*p values were calculated from Chi-Square tests; statistical significance is indicated using bold font

### Associations between standardized percent mammographic density change and breast cancer risk among the study population overall and by BBD histology

Compared with women with a minimal decrease in MBD, a protective association was observed among those who had an MBD decrease of greater than or equal to 5% per 2 years during follow-up (OR 0.64; 95% CI 0.38, 1.07), though this was not statistically significant (*p*-trend = 0.09) (Table 2). Similarly, non-statistically significant protective associations were observed for MBD decline of greater than 5% per 2 years irrespective of BBD histological classification. Additionally, no difference in breast cancer risk was observed for those who had stable/any increase in their MBD compared to women with minimal decrease of less than 5% MBD change per 2 years, among the overall BBD population (OR 1.09; 95% CI 0.68, 1.75) or by BBD histology. For the associations examined above, no interactions of MBD change with follow-up time or BMI were observed (Table 2). Positive associations for both baseline and follow-up mammograms and breast cancer risk overall and by BBD diagnosis were observed and are shown in Additional file 1: Table S1.

**Table 2:** Associations between standardized percent mammographic breast density change and breast cancer risk, overall and by BBD histological classification

Standardized MBD change (%, per 2 years)	Case *n* = 261	Control *n* = 249	*p*-value*	Multivariable Model	*p*-trend†
	*N* (%)	*N* (%)		OR (95 CI)†	
*BBD overall (N = 510)*					
Stable/Increase ≥ 0%	65 (24.9)	65 (26.1)	0.47	1.09 (0.68, 1.75)	
Minimal decrease less than 5%	151 (57.9)	132 (53.0)		1.00 (Referent)	
Decrease more than 5%	45 (17.1)	52 (20.9)		0.64 (0.38, 1.07)	
Trend				0.77 (0.57, 1.04)	0.09
Continuous				0.99 (0.95, 1.02)	0.46
*Non-proliferative diagnosis (N = 329)*					
Stable/Increase ≥ 0%	40 (26.5)	43 (24.2)	0.8	1.28 (0.71, 2.30)	
Minimal decrease less than 5%	85 (56.3)	100 (56.2)		1.00 (Referent)	
Decrease more than 5%	26 (17.2)	35 (19.7)		0.75 (0.39, 1.44)	
Trend				0.77 (0.53, 1.11)	0.16
Continuous				0.96 (0.92, 1.01)	0.13
*Proliferative diagnosis (N = 181)*					
Stable/Increase ≥ 0%	25 (22.7)	22 (31.0)	0.14	0.88 (0.37, 2.07)	
Minimal decrease less than 5%	66 (60.0)	32 (45.1)		1.00 (Referent)	
Decrease more than 5%	19 (17.3)	17 (23.9)		0.49 (0.20, 1.18)	
Trend				0.75 (0.44, 1.29)	0.30
Continuous				1.03 (0.96, 1.09)	0.41

No interactions of follow-up time or BMI with percent density change (standard) after adjusting for matching factors, BMI, and baseline density (quartiles) were observed

Standardized change in percent mammographic density (i.e., percent density change per two years) was calculated using percent density change from baseline to follow-up mammogram and divided by time intervals between two mammogram exams (percent density at follow-up mammogram − percent density at baseline mammogram)/(calendar years between baseline mammogram and follow-up mammogram/2)

Missing values in breast cancer risk factors were imputed using multiple imputations. Result estimates were calculated in five imputed datasets, separately, and averaged using statistical procedures

*BBD* benign breast disease, *BMI* body mass index, *CI* confidence interval, *OR* odds ratio

*p values were calculated from Chi-Square tests

^†^Point estimates and 95% confidence interval were determined from multivariable unconditional logistic regression models adjusting for continuous age at BBD and follow-up time between BBD and breast cancer diagnosis, BBD calendar year, baseline percent density (quartiles), and BMI

### Associations between standardized percent mammographic density change and breast cancer risk by age at BBD diagnosis and BBD histology

Among women < 50 years, no clear patterns of associations were observed between MBD change and risk of breast cancer (Table 3). However, among women ≥ 50 years at BBD diagnosis, a reduced risk of breast cancer was observed in women who had an MBD decrease of greater than 5% (OR 0.48; 95% CI 0.25, 0.92; *p*-trend = 0.03) as compared with women with a minimal decrease of less than 5% MBD change per 2 years. Upon further stratification by BBD histology, the protective association for MBD decline of more than 5% was most apparent for women who had a BBD diagnosis of proliferative disease (OR 0.32; 95% CI 0.11, 0.99); findings for those with non-proliferative disease were weaker (OR 0.70; 95% CI 0.30, 1.64) (Table 3). Similar but weaker patterns were observed when analyses were restricted to postmenopausal women (Additional file 1: Table S2).

**Table 3:** Associations between standardized percent mammographic breast density change and breast cancer risk, by age at BBD diagnosis and BBD histological classification

Standardized MBD change (%, per 2 years)	Age at BBD < 50 year (*n* = 173)					Age at BBD ≥ 50 year (*n* = 337)						*P*-interaction‡
	Case *n* = 94	Control *n* = 79	*p*-value*	Multivariable Model	*P*-trend†	Standardized MBD change (%, per 2 years)	Case *N* = 167	Control *N* = 170	*P*-value*	Multivariable Model	*p*-trend†	
	*N* (%)	*N* (%)		OR (95 CI)†			*N* (%)	*N* (%)		OR (95 CI)†		
Ov*erall (N = 173)*						*Overall (N = 337)*						
Stable/Increase ≥ 0%	13 (13.8)	13 (16.5)	0.77	0.79 (0.31, 2.01)		Increase ≥ 0%	52 (31.1)	52 (30.6)	0.21	1.17 (0.67, 2.05)		0.25
Minimal decrease less than 5%	62 (66.0)	53 (67.1)		1.00 (Referent)		Decrease less than 5%	89 (53.3)	79 (46.5)		1.00 (Referent)		
Decrease more than 5%	19 (20.2)	13 (16.5)		1.10 (0.45, 2.70)		Decrease more than 5%	26 (15.6)	39 (22.9)		**0.48 (0.25, 0.92)**		
Trend				1.18 (0.68, 2.06)	0.56	Trend				**0.66 (0.46, 0.96)**	**0.028**	
Continuous				0.99 (0.92, 1.06)	0.73	Continuous				0.98 (0.94, 1.03)	0.46	
*Non-proliferative diagnosis (N = 127)*						*Non-proliferative diagnosis (N = 202)*						
Stable/Increase ≥ 0%	9 (14.3)	11 (17.2)	0.82	0.87 (0.30, 2.56)		Increase ≥ 0%	31 (35.2)	32 (28.1)	0.44	1.43 (0.69, 2.93)		0.43
Minimal decrease less than 5%	43 (68.3)	44 (68.8)		1.00 (Referent)		Decrease less than 5%	42 (47.7)	56 (49.1)		1.00 (Referent)		
Decrease more than 5%	11 (17.5)	9 (14.1)		1.07 (0.36, 3.21)		Decrease more than 5%	15 (17.1)	26 (22.8)		0.70 (0.30, 1.64)		
Trend				1.11 (0.58, 2.15)	0.75	Trend				0.70 (0.44, 1.12)	0.14	
Continuous				0.97 (0.88, 1.06)	0.48	Continuous				0.97 (0.92, 1.03)	0.35	
*Proliferative diagnosis (N = 46)*						*Proliferative diagnosis (N = 135)*						
Stable/Increase ≥ 0%	4 (12.9)	2 (13.3)	–	–		Increase ≥ 0%	21 (26.6)	20 (35.7)	0.099	0.95 (0.36, 2.52)		0.64
Minimal decrease less than 5%	19 (61.3)	9 (60.0)		–		Decrease less than 5%	47 (59.5)	23 (41.1)		1.00 (Referent)		
Decrease more than 5%	8 (25.8)	4 (26.7)		–		Decrease more than 5%	11 (13.9)	13 (23.2)		**0.32 (0.11, 0.99)**		
Trend					–	Trend				0.61 (0.32, 1.20)	0.15	
Continuous					–	Continuous				1.02 (0.95, 1.09)	0.67	

Standardized change in percent mammographic density (i.e., percent density change per two years) was calculated using percent density change from baseline to follow-up mammogram and divided by time intervals between two mammogram exams (percent density at follow-up mammogram − percent density at baseline mammogram)/(calendar years between baseline mammogram and follow-up mammogram/2). Missing values in breast cancer risk factors were imputed using multiple imputations. Result estimates were calculated in five imputed datasets, separately, and averaged using statistical procedures

*BBD* benign breast disease, *BMI* body mass index, *CI* confidence interval, *MBD* mammographic breast density, *OR* odds ratio;

*P values were calculated from Chi-Square tests; statistical significance is indicated using bold font. *P *value was not calculated for proliferative diagnosis among women age at BBD < 50 years due to small cell counts

^†^Point estimates and 95% confidence interval were determined from multivariable unconditional logistic regression models adjusting for continuous age at BBD and follow-up time between BBD and breast cancer diagnosis, BBD calendar year, baseline percent density (quartiles), and BMI

^‡^*p* values for interactions of age at BBD (< 50 vs. ≥ 50 year) with percent density change (standard) after adjusting for matching factors, BMI, and baseline density (quartiles)

To determine whether associations between MBD decline and risk were modified by tissue composition of the BBD biopsies, associations between MBD change and breast cancer risk were examined stratified by histological tissue composition metrics (Additional file 1: Table S3). The inverse association with MBD decline and breast cancer risk was strongest among women who were ≥ 50 years at BBD diagnosis with high epithelium-to-stroma proportion (OR 0.35; 95% CI 0.14, 0.92, Additional file 1: Table S3). Similar associations were observed for high individual epithelial and stromal tissue for women ≥ 50 years but not among women < 50 years (Additional file 1: Table S3).

### Sensitivity analysis

Observed patterns of association were similar between the conditional and unconditional models restricted to matched case-control pairs for all pairs and stratified by age (Additional file 1: Table S4). In an additional sensitivity analysis to examine the potential impact of mammogram timing on the study findings, we repeated the analysis in Table 2 adjusting models for the time between mammogram collection and BBD diagnosis. The model results were similar to the main findings (data not shown).

## Discussion

In this study of women with BBD, we examined associations between temporal changes in MBD and risk of subsequent invasive breast cancer after a diagnosis of BBD. Overall, we found that women aged ≥ 50 years at the time of BBD diagnosis who experienced a subsequent decrease in their MBD had a reduced risk of breast cancer compared to women who had a minimal decrease. Further, among women aged ≥ 50 years, this risk reduction remained for those diagnosed with proliferative BBD, but not among women who had a non-proliferative BBD diagnosis. We also observed that stable/any increase, defined as no change or any increase found per 2 years in MBD, was not associated with a change in risk compared to those who experienced minimal MBD declines. Our findings suggest that decreasing MBD among women ≥ 50 years old with proliferative BBD, a BBD subgroup at higher risk of breast cancer, may be useful in reducing future breast cancer risk for this subgroup, for whom breast cancer prevention strategies are needed. Further, evaluating MBD decline among women with BBD may provide a helpful indicator for monitoring future breast cancer risk.

While much research has been carried out examining the associations between MBD and breast cancer risk, fewer studies have explored associations of temporal changes in MBD with risk of breast cancer among subgroups of women at higher risk. A recent systematic review and meta-analysis by Mokhtary and colleagues reviewed studies of MBD change and breast cancer risk [26]. With 9 studies eligible for inclusion, the meta-analysis examined associations between increased and decreased MBD over time and risk of breast cancer. A pooled analysis of four cohort studies found increasing MBD was associated with increased breast cancer risk [26]. Further, decreasing MBD was associated with reduced breast cancer risk [26]. Among the studies included was a prospective cohort study of women that participated in screening between 1993 and 2003 conducted by Kerlikowske and colleagues who observed a significantly lower breast cancer risk among women whose MBD decreased compared with women whose MBD remained stable over a median follow-up time of 3.2 years between mammograms [16] and a smaller study by van Gils [19] within the Nijmegen breast cancer screening program also suggested that MBD decline, measured over 10 years, was associated with reduced breast cancer risk. Further, more recent findings from Jiang and colleagues found that the rate of MBD decline over time was slower within the breast that developed breast cancer compared to controls within the nested case–control cohort study from the Joanne Knight Breast Health Cohort [20]. Conversely, Azam and colleagues, using the KARMA (Karolinska Mammography Project for Risk Prediction of Breast Cancer) cohort, a prospective screening cohort of Swedish women aged 30–79 years, showed no association between MBD change and risk of breast cancer during follow-up of 5.4 years between screens [15]. They also found no difference in breast cancer risk for women who experienced stable or increasing MBD over time compared to decreasing MBD and breast cancer risk, suggesting that annual changes in MBD do not influence short-term risk [15]. Their findings were in agreement with a prior case–control study by Vachon et al [18] and a nested case–control study within the Multiethnic Cohort conducted by Maskarinec and colleagues [17]. The reasons for the contrasting findings across these studies are not clear, but likely reflect differences in study populations, MBD assessment measures, definitions of MBD change, follow-up time and sample sizes. These prior studies conducted to date, however, have largely focused on the general screened population, and it has been unclear whether MBD changes relate to subsequent breast cancer risk among patients undergoing clinically indicated breast biopsies or among those with a diagnosis of BBD. Our study builds on this literature, extending the prior investigations to women diagnosed with BBD, and suggests that MBD change may be informative in the setting of BBD.

In line with what would be expected, elevated MBD both at baseline and follow-up was associated with increased breast cancer risk among the population overall and stratified by age, though not all statistically significant. These associations were stronger among women with proliferative disease, and particularly among women ≥ 50 years with a diagnosis of proliferative disease (Additional file 1: Table S1). The association in our analysis of declines in MBD and breast cancer risk observed among women ≥ 50 years at time of BBD diagnosis are likely due to the influence of multiple factors. Of note, this analysis did not account for post-BBD surveillance screening strategies or occurrence of further benign lesions that could have occurred among the study population. Further, age- and BBD-related differences with respect to the incidence and biology of breast cancer subtypes may contribute to our findings. While breast cancers occurring among younger women are more likely to be aggressive, ER-negative subtypes, those occurring among older women tend to be less aggressive and mostly ER-positive [27]. Proliferative changes on histology are early lesions that occur within the sequence of epithelial changes that culminate in the emergence of mostly ER-positive breast cancers [28]. Indeed, most of the cancers arising in this BBD population were ER + and low-stage [25], suggesting a potential role of hormone-dependent mechanisms in the etiopathogenesis of BBD-related breast cancer. It is conceivable that our observed associations between MBD decline and reduced risk of invasive breast cancer among older women with proliferative BBD may reflect shared hormone-dependent mechanisms underpinning MBD reduction and tumor development among this subset of women. Additionally, it is important to consider the associations among women who were < 50 years at time of BBD diagnosis and became post-menopausal at time of breast cancer diagnosis. Though information about menopausal status was not available at follow-up, it is likely that a proportion of this group underwent menopausal transition during follow-up, which may have also influenced MBD change and breast cancer risk. Studies that have explored relationships between the stages of menopausal transition and MBD change have mainly been focused on cancer free women. Engmann and colleagues showed within a longitudinal analysis, an annual decline in dense volume of 2.2 cm^3^ across menopausal transition. They also found that higher dense volume among premenopausal women was a strong predictor of annual declines during menopausal transition and that these associations were not influenced by other breast cancer risk factors [29]. Within our study, to account for this potential impact we adjusted the analysis for baseline density. However, to our knowledge, there have been no studies that have examined MBD change using serial imaging across the stages of menopause among the higher risk population of women with BBD. We were also unable to account for weight change in our analysis. BMI has previously been identified as a determinant of MBD change [15]. Similarly, to our knowledge, this has not been examined within a BBD population; however, few studies have examined these associations in cancer free women. For example, in a study by Hart and colleagues using data from the San Francisco Mammography Registry, their findings showed a longitudinal increase in BMI being associated with corresponding declines in percent dense volume [30]. However, the impact of these changes on breast cancer risk is not fully disentangled given the increased associations that have been described between weight gain and breast cancer risk [31, 32]. While the protective effect of MBD decline could be countered by weight gain, we were unable to assess this in the current study. Nevertheless, it is unlikely that post-BBD diagnosis weight gain, or loss, could have been differential by case–control status in the current study. Further studies specifically accounting for temporal changes in BMI would be required to conclusively determine its impact on MBD decline and subsequent invasive breast cancer risk among women with BBD.

Currently, the biological mechanisms that underlie MBD change are not fully defined. However, it is generally accepted that MBD reflects the underlying stromal and epithelial tissue components of the breast [5, 33]. This nested case–control study provides a further biological insight into the molecular correlates of MBD change in women with BBD, outside the setting of tamoxifen associated MBD decline. Our prior studies within this cohort have shown that high ESP is an independent marker of breast cancer risk among women with BBD [8]. Further, those prior analyses showed that women with combined high ESP and high MBD are at higher breast cancer risk compared with those with low ESP and low MBD, and these findings were stronger for women with non-proliferative BBD [8]. Building on that work within this current study, although limited by sample size, we suggest that temporal reductions in MBD were associated with reduced breast cancer risk even among high-risk women with elevated ESP, and especially among those > 50 years. Elevated ESP may represent a high-risk, but dynamic, tissue microenvironment susceptible to temporal changes that are reflected radiologically as decreased MBD. However, further studies integrating serial radiologic images with histological specimens are needed to characterize the relationships between temporal changes in MBD and ESP.

Strengths of this study include the case–control design within a well-defined longitudinal cohort of patients, with detailed information on BBD characteristics, risk factors and long-term follow-up. The availability of baseline and follow-up mammograms as well as archival tissue blocks is another important strength of this study. A key limitation of this study was the lack of diversity among the study population, which was mostly composed of white women. Given the differences in the distribution of breast density across race and ethnicity [34], it is important to understand if similar patterns are observed among women with BBD of non-white ethnicity. This will help to contextualize the potential clinical benefit of monitoring MBD over time for all women with BBD. This analysis was also constrained by limited sample size, which precluded detailed investigations of more refined strata of MBD change. For example, we combined women who experienced increases in their MBD and those whose MBD remained stable into one category. We did this to due to small sample sizes for the individual categories and because both scenarios constitute departures from the expected age-related MBD decline that occurs as part of the normal physiological processes during aging. Nonetheless, the clinical significance of this group warrants further investigation in future studies. The limited sample size also precluded detailed investigations of MBD among patients whose BBD and breast cancer were diagnosed in the ipsilateral breast. The absence of risk factor information over the course of study follow-up and at the time of breast cancer diagnosis was another important limitation of this study. For example, MBD is strongly inversely associated with BMI, and while our analyses controlled for baseline BMI, we did not have information on BMI at the time of second mammogram and therefore could not account for weight change over the course of follow-up. In addition, MBD is associated with MHT and information on MHT was limited to use at baseline and we were unable to account for any change in MHT use over the course of follow-up.

Challenges remain to accurately identify women with BBD who may benefit from tailored strategies, such as tamoxifen chemoprevention [35, 36], to reduce future risk of breast cancer. Existing breast cancer risk prediction tools, which aim to determine individual risk, typically include information on whether a previous biopsy was conducted and on a previous diagnosis of atypical hyperplasia or BBD [37]. Despite the continued refinement of these risk models to include an expanded panel of risk factors, their discriminatory power remains limited [37]. Many breast cancer risk models have been revised to incorporate MBD information and a recent systematic review that included seven studies showed statistically significantly improved discriminatory accuracy [38] suggesting that the inclusion of MBD in risk prediction models could improve performance. However, to date, investigations of the potential benefit of incorporating measures of MBD change into a breast cancer risk prediction model have been limited [39]. Using the Breast Cancer Surveillance Consortium (BCSC) model, Kerlikowske and colleagues found that the inclusion of MBD information at different time-points showed marginal improvement in discriminatory accuracy over the inclusion of MBD at one time point defined by BI-RADS. Their study focused on data from screening mammography registries and future studies are needed to expand these investigations to risk assessment for BBD patients that includes quantitative measures of MBD and its temporal changes.

In summary, we observed that temporal declines in MBD were associated with reduced risk of subsequent breast cancer among women with BBD aged ≥ 50 years, particularly among women with proliferative BBD. These findings suggest that monitoring MBD among this group may be a useful tool in determining risk and that strategies to actively reduce MBD may be helpful in reducing breast cancer risk among higher-risk BBD patients.

### Supplementary Information

**Additional file 1**. **Table S1**: Associations between baseline and follow-up percent mammographic density with breast cancer risk, overall and stratified by age at BBD and BBD histology. **Table S2**: Associations between standardized change in percent mammographic density and breast cancer risk among postmenopausal women. **Table S3**: Associations between standardized change in percent mammographic density and breast cancer risk, by histologic tissue composition of the diagnostic benign breast disease biopsy. **Table S4**: Associations between percent mammographic density change (standard) and breast cancer risk in overall women and by BBD histology among matched case–control sets using conditional logistic regression.

## Data Availability

The data that support these findings are not publicly available because they contain information that could compromise research participant privacy and confidentiality. The authors will make the data available upon reasonable request and with the permission of the Kaiser Permanente Center for Health Research in Portland, Oregon.

## Abbreviations

AFLB: Age at first live birth
BBD: Benign breast disease
BMI: Body mass index
ER: Estrogen receptor
H&E: Hematoxylin and eosin
KPNW: Kaiser Permanente Northwest
MBD: Mammographic breast density
MHT: Menopausal hormone therapy
OR: Odds ratio
