# Supplementary material for: Temporal changes in mammographic breast density and breast cancer risk among women with benign breast disease
Source: Breast Cancer Res. 2024 Mar 26;26:52. doi: 10.1186/s13058-024-01764-2 (PMC10967105; doi:10.1186/s13058-024-01764-2)
Supplement: Supplementary file 1 — Additional file 1. Table S1: Associations between baseline and follow-up percent mammographic density with breast cancer risk, overall and stratified by age at BBD and BBD histology. Table S2: Associations between standardized change in percent mammographic density and breast cancer risk among postmenopausal women. Table S3: Associations between standardized change in percent mammographic density and breast cancer risk, by histologic tissue composition of the diagnostic benign breast disease biopsy. Table S4: Associations between percent mammographic density change (standard) and breast cancer risk in overall women and by BBD histology among matched case–control sets using conditional logistic regression. [file 13058_2024_1764_MOESM1_ESM.docx]

Supplementary Table 1. Associations between baseline and follow-up percent mammographic density with breast cancer risk, overall and stratified by age at BBD and BBD histology.

Supplementary Table 2. Associations between standardized change in percent mammographic density and breast cancer risk among postmenopausal women.

Supplementary Table 3. Associations between standardized change in percent mammographic density and breast cancer risk, by histologic tissue composition of the diagnostic benign breast disease biopsy.

Supplementary Table 4. Associations between percent mammographic density change (standard) and breast cancer risk in overall women and by BBD histology among matched case-control sets using conditional logistic regression.
